# 

**Published:** 1850-10

**Authors:** 


### NOTICE TO CORRESPONDENTS.

Communications and Books for notice should be addressed to the Editor, care of Messrs. Lindsay & Blakiston.

Letters, &c., connected with the *business affairs* of the Journal should be addressed to the Publishers.

Papers for publication must be received *before* the 20th of the month, or they cannot appear in the forthcoming number.

The following Journals have been received in exchange:

New York Medical Gazette. Weekly.
The Boston Medical and Surgical Journal. (Weekly, Boston.)
Buffalo Journal.
Medical News.
Western Lancet.
North Western Medical and Surgical Journal. Sept.
Nordamericanischer Monats bericht für Natur-und Keilkunde.
The British American Journal of Medical and Physical Science. (Montreal.)
The London Lancet. (Weekly, London.)
The Medical Times. Weekly, London.
Dublin Medical Press.
Provincial Medical and Surgical Journal.
Edinburgh Monthly Journal. August and September.
Edinburgh Medical and Surgical Journal.
Dublin Quarterly Journal. August.
London Medical Gazette. May, July, August.
Pharmaceutical Journal.
London Journal of Medicine. July, August, September.
Gazette des Hopitaux. January to July inclusive.
Gazette Médicale. " "
Revue Medico-Chirurgicale.

---

The following works have also been received for notice:

Copland on Apoplexy and Palsy. From Lea & Blanchard.
Howard on the Eye.
Spectacles, their Uses and Abuses. By Sichel.
Eberle and Mitchell on Children. From Lippincott & Grambo.
Observations on certain of the Diseases of Young Children. By C. D. Meigs, M. D. From Lea & Blanchard.
Fownes' Chemistry for Students. 3d edition. From Lea & Blanchard.
A brief history of the existing controversy on the subject of Assimilated Rank in the United States Navy.

---

Communications have been received from:—

W. J. Reese, M. D., Alabama.
William Waters, M. D., Fredericktown, Md.
G. H. H. Koontz, Woodstock, Va.
E. C. Banks, Lawrenceville, Ill.
S. S. Hornor, Philadelphia.
J. M. Steiner, M. D., U. S. Army.
J. Travis, M. D., Tennessee.

---

The foreign correspondents of the Examiner will please direct their Exchanges and other communications to the care of Mr. Charles J. Skeet, 27 King William St., Charing Cross, London, or Mr. H. Bossange, 21 Bis, Quai Voltaire, Paris.

